# Coaxial technique-promoted diagnostic accuracy of CT-guided percutaneous cutting needle biopsy for small and deep lung lesions

**DOI:** 10.1371/journal.pone.0192920

**Published:** 2018-02-15

**Authors:** Lu Zhang, Lei Shi, Zhiping Xiao, Hong Qiu, Ping Peng, Mingsheng Zhang

**Affiliations:** The Department of Oncology, Tongji Hospital, Tongji Medical College, Huazhong University of Science and Technology, Wuhan, Hubei, China; A.C. Camargo Cancer Center, BRAZIL

## Abstract

Coaxial technique is extensively applied to facilitate percutaneous lung lesion biopsy. However, the impact of coaxial technique on diagnostic accuracy remains undecided. We reviewed 485 patients who underwent percutaneous CT-guided needle biopsies of lung lesions in our hospital. All of these biopsies were performed using either a cutting needle alone (n = 268) or a cutting needle combined with a coaxial needle (n = 217). The diagnostic accuracy and complications resulting from the two techniques were then compared. The diagnostic accuracies of the two techniques were comparably high, at 98.2% (with coaxial technique) and 95.9% (without coaxial technique), *p* = 0.24. Subgroup analysis discovered that for patients with lesions measuring < 1.5 cm and needle path length ≥ 4 cm, the coaxial technique achieved a higher diagnostic accuracy (95.5% vs. 72.7%, *p* = 0.023). The biopsy was well tolerated in all of the patients. Pneumothorax occurred less often in patients who were biopsied with the coaxial technique (19 versus 43, *p* = 0.024). Thus, the application of the coaxial technique could improve diagnostic accuracy in patients with small and deep lung lesions, and could reduce the risk of pneumothorax. The combined use of cutting needles with coaxial needles is the preferred technique for performing percutaneous CT-guided lung biopsies.

## Introduction

Percutaneous Computed Tomography (CT)-guided needle biopsy of lung lesions is a well-established and safe technique for the diagnosis of pulmonary nodules[1]. In earlier reports, fine-needle aspiration has been described as achieving most lung biopsies with a diagnostic specificity of nearly 100% and a sensitivity of over 90% [2, 3]. However, the diagnostic accuracy of this technique in benign lung lesions was reported to be lower than 70% [3]. Compared with fine-needle aspiration, tissue core biopsy with a cutting needle achieves a better diagnostic accuracy for benign lung lesions and a comparably high diagnostic accuracy for malignant lung lesions[3, 4] without increasing the risk of complications. With fine-needle aspiration, an on-site cytopathologic evaluation is often recommended, but with the cutting needle technique this evaluation can be omitted. Thus, the percutaneous CT-guided cutting needle biopsy has been accepted as the superior technique for the diagnosis of lung lesions[5].

Percutaneous lung lesion biopsy, either fine needle aspiration or tissue core biopsy, is often performed under the introduction of coaxial needle[6]. Coaxial technique makes it much easier to repeat sampling and obtain adequate specimens during percutaneous lung lesion biopsy, without increasing the number of passes through the pleura. Furthermore, the procedure duration can be remarkably shortened. However, since the diagnostic accuracy of cutting needle biopsy performed by an experienced radiologist is higher than 90%[3, 4, 7], it is questionable that if the application of coaxial technique could further promote the diagnostic accuracy of percutaneous lung lesion biopsy. An early study compared the diagnostic accuracy of percutaneous fine needle aspiration of lung lesions with or without coaxial needle[8]. They found that with the on-site cytopathologic evaluation, the coaxial technique didn’t improve the diagnostic accuracy. Recently, Nour-Eldin reported a retrospective analysis of patients received coaxial and non-coaxial CT guided lung biopsy[7]. The biopsy yield was more diagnostic and conclusive in the coaxial group (93.4%) in comparison to the non-coaxial group (87.7%). These discordant results manifest that the impact of coaxial technique on diagnostic accuracy remains undecided.

Pneumothorax is the most common complication resulting from percutaneous CT-guided lung biopsies. The risk factors include needle size, the number of needle insertions, the procedure duration, the depth from pleura to lesion, the angle of needle route, and emphysema[2, 9–14]. A coaxial needle is larger than a cutting needle, and thus it may add to the pleura injury. However, the application of coaxial needles avoids traversing the pleura repeatedly, and it shortens the procedure’s duration. Therefore, the effect of coaxial technique on pneumothorax rate can’t be presumed theoretically. Here, we report a retrospective study on two cohorts of patients who underwent CT-guided lung biopsies with either cutting needles alone or cutting needles combined with coaxial needles. The purpose of this study was to evaluate the accuracy and safety of the coaxial technique.

## Materials and methods

### Patients

This retrospective study was approved by the ethical committee of Tongji Hospital, Tongji Medical college, Huazhong University of Science and Technology (20140101). The study involved 485 patients (353 men and 132 women) who underwent percutaneous CT-guided lung biopsies in our institute from May 2011 to December 2013. All of the patients had pulmonary nodules (of diameters between 0.5 and 12 cm) as demonstrated by contrasted CT scans and with adequate coagulation status. Percutaneous CT-guided biopsies were performed once for each patient with cutting needles, with or without coaxial needles. Both biopsy methods were approved and routinely used in our institution. All biopsies were independently performed by one of two experienced operators (also listed as authors). Both operators had performed CT guided pulmonary biopsies for more than 4 years. The biopsy method was subjectively decided by the operator according to arbitrary principles. Approximately, the deep and small lesions are more difficultly to be approached, thus the operators might tend to choose coaxial technique for biopsies of such lesions. On the contrary, the superficial and big lesions are easier to be approached, thus the operators might decide biopsy method without any tendency. However, the cut-off of lesion size and depth were not explicitly defined in this study. The operators chose the biopsy method according to personal judgement and experience. The clinical data was retrospectively collected and analyzed in June and July, 2014. Patients biopsied with cutting needles combined with coaxial needles were assigned into coaxial group, and patients biopsied with cutting needles alone were assigned into non-coaxial group. Written informed consents were obtained before the procedures in all cases.

### Biopsy

Before the procedure, each patient’s previous contrasted CT scan was reviewed concerning the locations of their lesions and of adjacent important structures, to determine patient positioning for biopsy. The patients were placed on the examination bed in prone, supine, or lateral decubitus positions, and prebiopsy scans were taken by a single slice spiral-CT (CT/e, GE, Easton Turnpike Fairfield, State of Connecticut) with 3mm slice thickness. Next, optimal access was determined according to the principles of finding the most direct route, the shortest route length, and of avoiding the suspicious necrosis and cavity. The optimal skin entry site, needle angle, and route length was indicated through CT images. Local antisepsis and anesthesia were applied, followed by the biopsy procedure.

In coaxial group, the coaxial needles (17 Gauge Co-axial Introducer Needle, InterV-MDTech, Gainesville, Florida) were inserted towards the lesions as planned. For each patient, a second CT scan was acquired to ensure correct position of the needle. If necessary, the needle was adjusted to reach the lesion as confirmed by an additional CT scan. A biopsy needle (18 Gauge Biopince Full Core Biopsy Needle, InterV-MDTech, Gainesville, Florida) was introduced through the coaxial needle to reach the lesion, and then fired to obtain a tissue core of 2 cm length. The introduction and fire of biopsy needle was repeated three times to obtain three samples, as suggested by Wehrschuetz[15].

In non-coaxial group, the same procedure of insertion and position confirmation was performed with a biopsy needle (18 Gauge True-Core II Biopsy Instrument, InterV-MDTech, Gainesville, Florida) instead of a coaxial needle. The needle was then fired to obtain a tissue core of also 2 cm length. The procedure was repeated three times to obtain three samples, as with the patients in coaxial group.

After the biopsy procedures, all patients were observed closely for 10–30 min. To avoid unnecessary exposure to radiation, CT scans were performed only for patients with suspicious pneumothorax. Small asymptomatic pneumothoraxes were treated conservatively, with monitoring of vital signs and symptoms to confirm stability. Patients with stable pneumothorax were returned to the ward for further observation. A chest tube was inserted for drainage in patients who had pneumothorax with respiratory distress or shortness of breath.

### Assessment of diagnostic yield

The biopsy diagnoses were classified into the following three categories: malignant (including atypical adenomatous hyperplasia), benign, and nondiagnostic. The results were considered nondiagnostic if the procedure was terminated before a specimen was obtained, or if the specimens obtained were inadequate for diagnosis. Diagnoses of malignant and benign disease were considered as positive and negative results, respectively.

Positive biopsy results were further classified as true-positive or false-positive if the final diagnoses were of malignant or benign disease, respectively. Negative biopsy results were further classified as true-negative or false-negative if the final diagnoses were of benign or malignant disease, respectively. All patients were followed up to determine their final diagnoses. The final diagnoses were determined by surgical specimens in patients who underwent surgery. In patients who did not undergo surgery, the final diagnosis was determined to be malignant or benign disease according to the postprocedural clinical course (e.g., increased or stable lesion size, lesion regression by chemotherapy or antibiotics, or presence of metastasis).

The diagnostic accuracy was calculated using the following formula: diagnostic accuracy (%) = number of patients truly diagnosed (true positive + true negative) / total number of patients.

### Statistical analysis

Quantitative variables were compared using a *t*-test, performed using a Student’s *t*-test and a Mann–Whitney test for variables with and without a normal distribution, respectively. Qualitative variables were compared using Pearson’s chi-square coefficient test. A *p* value of < 0.05 was considered to be statistically significant.

## Results

A total of 485 patients were analyzed: 217 in coaxial group, and 268 in non-coaxial group. The features of the two groups are summarized in Table 1. The populations of the two groups were similar in terms of age, sex and cavitation. On average, patients in coaxial group had smaller and deeper lesions, and shorter procedure durations than those in non-coaxial group.

**Table 1:** Comparison of patients, lesions, and procedural variables in both groups.

	Coaxial group *n* = 217	Non-coaxial Group *n* = 268	*p* value
Sex (*n*)			
men	162	191	0.46
women	55	77	
Age (years)			
Mean ± SD	45 ± 12.5	44 ± 13.9	0.18
Range	31–78	35–74	
Lesion location			
Right upper lobe	67	65	0.13
Right middle lobe	31	38	0.97
Right lower lobe	46	55	0.94
Left upper lobe	36	51	0.56
Left lower lobe	37	59	0.21
Long lesion diameter (cm)			
Mean ± SD	3.9 ± 1.6	5.3 ± 2.5	< 0.0001
Range	0.7–8.4	1.4–12	
Short lesion diameter (cm)			
Mean ± SD	3.1 ± 1.4	3.7 ± 1.8	0.00008
Range	0.5–6.3	1–8.3	
Lesion depth (cm)			
Mean ± SD	5.9 ± 1.4	3.2 ± 1	< 0.0001
Range	2.6–8.6	1.3–6.5	
Necrosis or cavitation (*n*)	16	19	0.96
Procedure duration (min)			
Mean ± SD	8.4 ± 1.7	22.7 ± 3.4	< 0.0001
Patient position			
Supine	106	135	0.088
Prone	84	122	
Lateral	27	11	
Specimen number (*n*)			
Mean± SD	3 ± 0	2.3 ± 0.9	0.055
Specimen number* (*n*)			
Mean± SD	3 ± 0	1.4 ± 0.6	< 0.0001

*: The subgroup with lesions measuring < 1.5 cm and needle path> 4 cm.

The diagnostic yields of the two groups are shown in Table 2. The diagnostic accuracy for coaxial group and non-coaxial group were 98.2% and 95.9%, respectively. There was no significant difference between the two groups (*p* = 0.24). In coaxial group there were 4 diagnosis failures, of which all were false negative cases. In non-coaxial group there were 11 diagnosis failures, including 6 false negative and 5 nondiagnostic cases. 2 false negative patients in coaxial group and 1 false negative patient in non-coaxial group underwent surgery and turned out to be malignancies. All of the nondiagnostic cases were caused by insufficient collection of material. The histopathologic results are shown in Table 3.

**Table 2:** The diagnostic yields of the two groups.

	Coaxial group *n* = 217	Non-coaxial Group *n* = 268
True positive (*n*)	200	233
True negative (*n*)	13	24
False positive (*n*)	0	0
False negative (*n*)	4	6
Nondiagnostic (*n*)	0	5
Diagnostic accuracy	98.2%	95.9%

**Table 3:** The histopathologic results of the two groups.

	Coaxial group *n* = 217	Non-coaxial Group *n* = 268
Lung adenocarcinoma	52	77
Lung squamous cell carcinoma	50	68
NSCLC	17	27
Small cell carcinoma	44	25
Metastatic malignancy	37	36
Chronic inflammation	5 (3 false negative)	5 (1 false negative)
Tuberculosis	4	4
Fibrosis	2	1 (false negative)
Granuloma	2 (1 false negative)	7 (4 false negative)
Lung adenoma	1	0
Hamartoma	1	0
Fungus infection	1	2
Abscess	1	5
Infection	0	5
Pulmonary atelectasis	0	1
Nondiagnostic	0	5

NSCLC: non-small cell lung caner

The diagnostic yields were further analyzed in subgroups according to lesion diameter (< 1.5 cm or ≥1.5 cm) or needle path length (< 4 cm or ≥ 4 cm), as shown in Table 4. In the subgroup with lesions measuring < 1.5 cm and the subgroup with needle path length ≥ 4 cm, the coaxial technique led to higher diagnostic accuracy, but the difference was not significant. Next, we focused on the subgroup with lesions measuring < 1.5 cm and needle path length ≥ 4 cm. In this subgroup, the diagnostic accuracy for coaxial group was significantly better than for non-coaxial group.

**Table 4:** The diagnostic yields in different subgroups.

		True positive (*n*)	True negative (*n*)	False positive (*n*)	False negative (*n*)	Non-diagnostic (*n*)	Diagnostic accuracy	*p* value
lesion diameter < 1.5 cm	Coaxial group *n* = 55	41	12	0	2	0	96.4%	0.11
	Non-coaxial Group *n* = 48	38	3	0	4	3	85.4%	
lesion diameter ≥ 1.5 cm	Coaxial group *n* = 162	159	1	0	2	0	98.8%	0.97
	Non-coaxial Group *n* = 220	195	21	0	2	2	98.2%	
needle path length < 4 cm	Coaxial group *n* = 35	32	3	0	0	0	100%	0.74
	Non-coaxial Group *n* = 195	177	13	0	3	2	97.4%	
needle path length ≥ 4 cm	Coaxial group *n* = 182	168	10	0	4	0	97.8%	0.059
	Non-coaxial Group *n* = 73	56	11	0	3	3	91.2%	
lesion diameter < 1.5 cm and needle path length ≥ 4 cm	Coaxial group *n* = 44	33	9	0	2	0	95.5%	0.023
	Non-coaxial Group *n* = 22	14	2	0	3	3	72.7%	

The biopsy was well tolerated in all of the patients. Complications included pneumothorax, haemoptysis and chest pain (Table 5). Pneumothorax occurred in 19 patients of coaxial group and 43 patients of non-coaxial group. This difference was significant. Only 11 patients needed chest tube placement: 3 in coaxial group and 8 in non-coaxial group. Haemoptysis occurred in 24 patients of coaxial group and 29 patients of non-coaxial group, but this condition was always alleviated within 72 h. Some of the patients in the study experienced chest pain following the procedure.

**Table 5:** The complications of both groups.

Complications (*n*)	Coaxial group *n* = 57	Non-coaxial Group *n* = 101	*p* value
Pneumothorax	19	43	0.024
Chest tube placement	3	8	0.383
Haemoptysis	24	29	0.950
Chest pain	11	21	0.299

## Discussion

Percutaneous CT-guided needle biopsy of lung lesions is a well-established and safe technique for the diagnosis of pulmonary nodules. The use of cutting needles, which can obtain tissue material for both diagnostic staining and genetic examination, is the preferred instrument for biopsies. The coaxial needle facilitates biopsies and has some theoretic advantages. In this study, we provide evidence that the coaxial technique increases diagnosis accuracy concerning small deep lesions and reduces the risk of pneumothorax, as compared with use of the cutting needle alone.

Various studies have shown that diagnostic accuracy is influenced by lesion size and depth. Small and deep lesions are difficult to approach due to respiratory movement and the increased likelihood of operation errors. Priola et al. found that the diagnostic accuracy was only 68% for lesions measuring < 1.5 cm[3]. Ohno et al. also reported that the diagnostic accuracy was 52% for lesions measuring ≤ 1.0 cm, and 74% for lesions measuring 1.1 to 1.5 cm. These researchers analyzed lesion depth as well, finding that the diagnostic accuracy for needle path lengths of 4 cm or less was significantly greater than that for lengths greater than 4 cm[16]. In this study, the diagnostic accuracy of both biopsy techniques was comparably high. In subgroup analysis, the coaxial technique achieved better diagnostic accuracy in patients with lesions measuring < 1.5 cm and those with needle paths > 4 cm. However, the differences in results were not significant for either of these subgroups. We then analyzed the subgroup with lesions measuring < 1.5 cm and needle path> 4 cm. In this subgroup, the diagnostic accuracy in the coaxial group was significantly better than for the control group. This difference may have resulted from differences in numbers of specimens collected. A retrospective analysis by Wehrschuetz indicated that diagnostic accuracy with one, two and three specimens was 63.6%, 89.2%, and 91.5%, respectively[15]. Beyond three specimens, additional biopsies did not show any higher impact on accuracy, and therefore Wehrschuetz recommended taking three specimens. Hiraki’s analysis of 1000 pulmonary lesion biopsies also indicated that the acquisition of two or fewer specimens was a significant independent risk factor for diagnostic failure[10]. In our study, three specimens were acquired without exception for patients in the coaxial group. But in the control group, the mean specimen number was 2.3, or even 1.4 in the subgroup with lesions measuring < 1.5 cm and needle path> 4 cm. The coaxial technique facilitated repeated sampling, and enabled the collection of adequate specimens; hence, a higher diagnostic accuracy was achieved. These results indicated that for the purpose of diagnosis, use of the cutting needle alone was adequate for biopsies of most lesions, but the coaxial technique could improve diagnostic accuracy for small and deep lesions.

Moreover, with the discovery of driver genes and the establishment of target therapy, nowadays the purpose of lung lesion biopsy is not just for diagnosis. 70% of pulmonary nodules of clinical significance were proved to be malignant tumors, either primary or metastatic[2, 10, 17]. In the scenario of non-small cell lung cancer (NSCLC), tissue samples are required not only for diagnosis, but also for the determination of genetic alterations which predict the efficiency of target drugs [18]. More importantly, such genetic alterations with therapeutic significance are still increasing and the corresponding target drugs are innovating the treatment of NSCLC[19]. Therefore, more tumor tissue samples are required for optimizing and individualizing of the treatment of NSCLC in the future. Biopsy using the coaxial technique is more likely to obtain adequate tissue samples for all the potential examinations.

Pneumothorax is the most common complication resulting from percutaneous CT-guided lung biopsies, and this problem is undoubtedly associated with needle diameter. The pneumothorax rate was 4% when using a 21-23G needle[2], 12–26.6% when using a 19G needle[11, 12], and 26% when using a 16G needle[13]. One previous study analyzed the pneumothorax rate when using biopsy needles of different sizes, and demonstrated that pneumothorax occurred more frequently when using 18G needles than when using 19G needles (*p* < 0.001)[14]. In our study, 17G coaxial needles were applied in the coaxial group, and 18G biopsy needles were applied in the control group. Nevertheless, the coaxial group achieved a lower pneumothorax rate than the control group, despite the larger needle diameter. This result may be attributed to fewer needle insertions and shorter procedure duration. Air leakage through the damaged visceral pleura is the main cause of pneumothorax in patients receiving percutaneous needle biopsies. Therefore, visceral pleura damage is the primary determinant of pneumothorax. The correlation between pneumothorax and the number of needle insertions has been repeatedly observed[11, 16]. The biopsy with the coaxial technique needed only one piercing of the pleura during the whole procedure, and thus diminishes pleura damage. Recently Nour-Eldin reported that the incidence of pneumothorax wasn’t significantly different between non-coaxial group and coaxial group who received CT-guided percutaneous lung biopsy[7]. However, the mean specimen number was 1.2 in non-coaxial group in this study, which means the number of needle insertion was similar in both groups. The discrepancy between Nour-Eldin’s and our results demonstrates that the application of coaxial technique could acquire more samples without increasing the risk of pneumothorax.

Procedure duration also affects the rate of pneumothorax rate[11]. The biopsy using the coaxial technique took less time, meaning that the needle stayed across the visceral pleura for a shorter time. Therefore, the procedure caused less pleural damage. Overall, the rate of pneumothorax following the percutaneous CT-guided lung biopsy was acceptable, and the coaxial technique further reduced the risk of pneumothorax.

However, this retrospective analysis has some limitations. The number of patients with small and deep lesions is small (44 and 22, respectively). The repeatability of the results needs to be validated in a larger series of patients. The subjective group assignment by the two operators may bias the results. Moreover, the diagnostic accuracy of CT guided pulmonary biopsies varies from operator to operator. The result of this study needs to be validated in patients biopsied by other operators. Therefore, a well-designed multi-center prospective randomized controlled clinical trial is needed to confirm the conclusions.

## Conclusions

The diagnostic accuracies of cutting needle biopsies with or without coaxial technique were comparably high in this study. The application of the coaxial technique could shorten procedure time and was associated with lower pneumothorax rate. Accordingly, coaxial technique was recommended for lung biopsy with cutting needle. Moreover, in patients with small and deep lesions, the diagnostic accuracy was unsatisfactory by cutting needle alone, but was improved with the application of the coaxial technique.

## Supporting information

S1 Table Data of coaxial group. (XLSX)

S2 Table Data of non-coaxial group. (XLSX)
